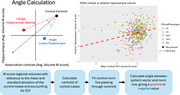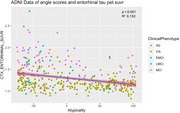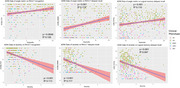# Developing a novel metric of atypicality in Alzheimer's Disease regional volume changes

**DOI:** 10.1002/alz70861_108954

**Published:** 2025-12-23

**Authors:** Briar Nowling, Hamsanandini Radhakrishnan, Christopher A Olm, Christopher A Brown, Sandhitsu R. Das, James C. Gee, Corey T. McMillan, Allison Snyder, Lauren Massimo, David A. Wolk, David J. Irwin, Jeffrey S Phillips

**Affiliations:** ^1^ Frontotemporal Degeneration Center, University of Pennsylvania, Philadelphia, PA USA; ^2^ Penn FTD Center, University of Pennsylvania, Philadelphia, PA USA; ^3^ Department of Neurology, University of Pennsylvania, Philadelphia, PA USA; ^4^ University of Pennsylvania, Philadelphia, PA USA; ^5^ Penn Image Computing and Science Laboratory (PICSL), University of Pennsylvania, Philadelphia, PA USA; ^6^ Penn Image Computing and Science Laboratory, Philadelphia, PA USA; ^7^ Center for Neurodegenerative Disease Research, Perelman School of Medicine, University of Pennsylvania, Philadelphia, PA USA; ^8^ Frontotemporal Degeneration Center, Department of Neurology, Perelman School of Medicine, University of Pennsylvania, Philadelphia, PA USA; ^9^ Penn Frontotemporal Degeneration Center, Department of Neurology, Perelman School of Medicine, University of Pennsylvania, Philadelphia, PA USA; ^10^ Department of Neurology, Perelman School of Medicine, University of Pennsylvania, Philadelphia, PA USA; ^11^ Penn Frontotemporal Degeneration Center, Department of Neurology, Perelman School of Medicine, University of Pennsylvania, Philadelphia, PA USA

## Abstract

**Background:**

Recent research on heterogeneity in Alzheimer’s disease (AD) highlights the need for imaging metrics that are equally sensitive in patients with atypical (limbic‐ or neocortical‐predominant) and typical patterns of degeneration. We developed an original model based on MRI volumetry that independently quantified disease atypicality and severity and related these metrics to clinical and imaging measures of AD in the ADNI repository.

**Method:**

In a dataset of 465 3T MRI scans (230 male; 264 cognitively normal (CN), 164 MCI, 37 AD), we computed regional volumes adjusting for intracranial volume for the neocortex (average of superior temporal, middle frontal, and inferior parietal cortices) and anterior hippocampus. We then plotted each individual’s hippocampal and neocortical atrophy as coordinates in a 2‐dimensional atrophy space (Figure 1). We used the centroids of CN and MCI/AD cases, respectively, to define a line representing the typical relationship between hippocampal and neocortical atrophy. For each individual, we defined a vector connecting their observed atrophy to the CN centroid. Atypicality was quantified by the angle between this vector and the typicality reference line: an individual whose vector lay above the typicality line had a positive angle, indicating hippocampal sparing; while a vector below the line had a negative angle, indicating limbic‐predominant atrophy. Individual disease severity was quantified by vector length. We evaluated this model using linear regression to test associations of atypicality and severity with tau PET uptake in entorhinal cortex; and with three memory measures: delayed recall on the logical memory test and Rey auditory verbal learning test (RAVLT) plus recognition on the RAVLT.

**Result:**

Both atypicality and severity were significant predictors of entorhinal tau PET uptake (Figure 2), RAVLT delayed recall, RAVLT recognition, and logical memory delayed recall (Figure 3; all *p* < 0.001). Severity had minimal association with atypicality (p = 0.0044, R^2^ = 0.018).

**Conclusion:**

A novel atypicality metric was related to both the burden of entorhinal tau and degree of memory impairment in ADNI cases, independently of severity. Future research will extend this model to atypical clinical syndromes and evaluate its ability to distinguish typical, limbic predominant, and hippocampal sparing cases.